# Development and pilot testing of PHARAO—a decision support system for pharmacological risk assessment in the elderly

**DOI:** 10.1007/s00228-017-2391-3

**Published:** 2017-12-02

**Authors:** Ylva Böttiger, Kari Laine, Tuomas Korhonen, Janne Lähdesmäki, Tero Shemeikka, Margaretha Julander, Maria Edlert, Marine L. Andersson

**Affiliations:** 1 Division of Drug Research, Department of Medical and Health Sciences, Linköping University, 581 83 Linköping, Sweden; 2 Medbase Ltd., Turku, Finland; 3 Department of Pharmacology, Drug Development and Therapeutics, University of Turku, Turku, Finland; 4 Division of Clinical Neurosciences, Turku University Hospital and University of Turku, Turku, Finland; 5 Department of E-health and Strategic IT, Stockholm County Council, Stockholm, Sweden; 6 Division of Clinical Pharmacology, Department of Laboratory Medicine, Karolinska Institutet, Karolinska University Hospital, 141 86 Stockholm, Sweden

**Keywords:** Adverse drug reaction, Clinical decision support system, Drug interactions, Polypharmacy, Elderly

## Abstract

**Purpose:**

The aims of this study are to describe the development of PHARAO (Pharmacological Risk Assessment Online), a decision support system providing a risk profile for adverse events, associated with combined effects of multiple medicines, and to present data from a pilot study, testing the use, functionality, and acceptance of the PHARAO system in a clinical setting.

**Methods:**

About 1400 substances were scored in relation to their risk to cause any of nine common and/or serious adverse effects. Algorithms for each adverse effect score were developed to create individual risk profiles from the patient’s list of medication. The system was tested and integrated to the electronic medical record, during a 4-month period in two geriatric wards and three primary healthcare centers, and a questionnaire was answered by the users before and after the test period.

**Results:**

A total of 732 substances were tagged with one or more of the nine risks, most commonly with the risk of sedation or seizures. During the pilot, the system was used 933 times in 871 patients. The most common signals generated by PHARAO in these patients were related to the risks of constipation, sedation, and bleeding. A majority of responders considered PHARAO easy to use and that it gives useful support in performing medication reviews.

**Conclusions:**

The PHARAO decision support system, designed as a complement to a database on drug-drug interactions used nationally, worked as intended and was appreciated by the users during a 4-month test period. Integration aspects need to be improved to minimize unnecessary signaling.

## Introduction

### Background

The number of drugs per patient has increased steadily over many years, especially in the elderly, as new treatment options have become available and patients live longer with chronic disease [1–4]. Even though the oldest part of the population is underrepresented in clinical trials, it can in many cases be assumed that the benefit of a given treatment, as measured by numbers needed to treat, increases with increasing age, in parallel to the increased risk of illness, as has been shown for, e.g., stroke prevention in atrial fibrillation [5]. However, with the increasing number of drugs follows an increased risk for unwanted adverse effects due to drug-drug interactions and additive pharmacological effects, leading to both patient suffering and healthcare costs [6–11]. Together, the need for a careful, patient-oriented risk-to-benefit analysis for each treatment increases with increasing age. Decision support systems are important tools to assist healthcare personnel, as well as patients themselves, in this work [12, 13].

### Previous experience of decision support systems

We have previously developed a drug-drug interaction database, Sfinx, to be integrated in computerized clinical decision support systems [14]. This system is now in use in most electronic health record systems in both Sweden and Finland, as well as in several other countries. To decrease the risk of alert fatigue, due to the system signaling too often, it has been an ambition from the start of the Sfinx project to have a restrictive approach to what drug pairs should be included. Still, the database contains more than 20,000 possibly interacting drug pairs (based on 1427 active substances).

### Rationale for the new decision support system

It has often been difficult to clearly define and act upon the limits on what interaction pairs to include into Sfinx. This is probably one of the main reasons for the great variation of content when comparing different drug-drug interaction databases [15–18]. We have also experienced a pedagogical challenge in explaining to prescribers the abilities, as well as the limits, of the database. Furthermore, for the prescriber, there is the problem of more than two drugs interacting, which will not be directly evident from any drug interaction database. To meet some of these challenges and as a complement to Sfinx, we have developed the PHARAO system for Pharmacological Risk Assessment Online. The PHARAO database contains a scoring of the risk of any of nine common and/or serious adverse effects for all substances also included in Sfinx, allowing for an evaluation of the overall risk from the patients’ complete medication list.

### The aim of the present work

We describe the development of the PHARAO system and present data from a pilot study, testing the use, functionality, and acceptance of PHARAO in a clinical setting. We, thus, hope to support not only the rational use of drugs, but also the rational use of decision support systems.

## Methods

### PHARAO development

The PHARAO project was developed and performed by the Sfinx working group, consisting of specialists in clinical pharmacology and neurology, pharmacists, and software developers. Specialists of other clinical disciplines, such as cardiology and nephrology, were consulted. The following nine pharmacological risks were included: risk of bleeding, sedation, orthostatism, constipation, anticholinergic side effects, serotonergic side effects, nephrotoxicity, QT prolongation/arrhythmia, and seizures. These properties were chosen with focus on adverse events relevant in the elderly patient. The choice was based on the clinical experiences in the Sfinx working group and the challenge of addressing pharmacodynamic effects in the format of pairwise drug interactions, as well as comments and questions received from end users of the Sfinx database. Thus, the aim was for the two systems to complement each other.

A standard set of literature sources for the evaluation and scoring of substances was determined, including pharmacological handbooks, summary of product characteristics, scientific evaluations available through EMA (European Medicines Agency), and relevant review articles retrieved through PubMed. All 1427 substances included in the Sfinx database at the time were scored from 0 (for no pharmacological effect) to 3 (for a strong pharmacological effect) for eight of the nine properties, according to a standard operating procedure. The scoring was principally based not only on clinical evidence of side effects, but also on pre-clinical and clinical evidences for receptor affinity [19]. For renal toxicity, there was only a score of 0 or 1, as we found no solid basis for grading the risk further. One person of the team scored one property for all substances, to get the best overall view. The scoring was then re-evaluated independently by two specialists in clinical pharmacology.

**Fig. 1:**
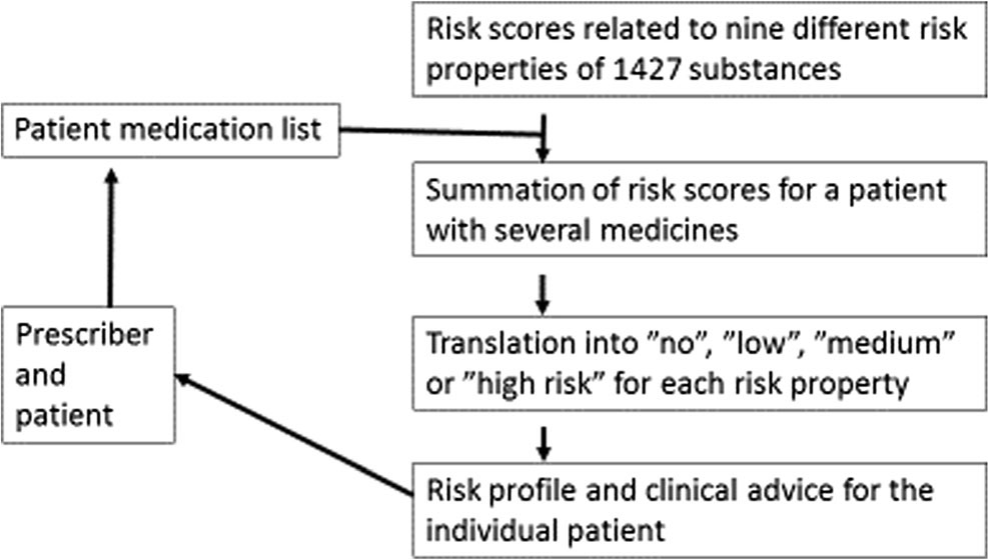
Workflow of the PHARAO system in relation to patient information input and output

For each of the properties, a literature search was performed to determine to what extent the combined risk resulting from two or more drugs administered together can be expected to be less than additive, additive, or synergistic and also whether the risk is considered to be dose-dependent. Algorithms for summing up the risk for each property from several substances were developed, based on the literature findings. The sum of scores is finally translated into an alert on low, medium, or high risk of adverse effects. The algorithms, including the cutoff levels for the degree of risk indicated, differ between the properties. These algorithms were later re-evaluated after being tested in relation to a set of anonymous patient medication lists. A set of standardized comments with consequence and recommendations in relation to each risk and risk level was agreed upon. The workflow of the decision support system is illustrated in Fig. 1.

### Pilot study

A pilot study of the PHARAO system was performed in two geriatric clinics/wards and three primary healthcare centers during a 4-month period, from October 2014 to the end of January 2015. Email addresses to a total of 129 physicians (all that were currently employed at the five units, with approximately half in primary health care) were supplied by the head of departments. All units used the same electronic health record (TakeCare), including recordings and prescriptions of medicines. The PHARAO system was made available to the physicians via a toolbar (the Janus toolbar), already integrated into the electronic health record, with “buttons” signaling in color if there is safety information to be retrieved for the individual patient. Apart from the new PHARAO button, the toolbar already included buttons signaling for drug-drug interactions (Sfinx), dosing recommendations according to renal function, and warnings related to unsuitable drugs for the elderly. The toolbar is visible as soon as the medication list is opened or a new prescription is issued, but there are no pop-up alerts. Participating physicians were informed about the study and the availability of the PHARAO system and had a short introduction to its functions but were not obliged to use it. Before the study period, an electronic questionnaire was sent by email to all physicians with questions regarding their pre-study conception of pharmacological risks and their expectations on the tool.

During the study period, all use of the PHARAO system was registered with regard to the signals generated, as well as sex, age, and the medication list of the patient. After the study period, a second questionnaire was sent to the same email addresses with questions regarding the use and evaluation of PHARAO.

## Results

### Development of the PHARAO system

The number of substances scored for the different pharmacological properties is shown in Table 1. Sedation is the risk that was connected to the largest number of substances, followed by risk of seizures and constipation. A total of 732 drugs were considered to possess one or more of the nine given risk properties, whereas 695 drugs had none of the risks.

**Table 1:** Number of substances, among a total of 1427, classified for the different pharmacological properties

	1	2	3	1 to 3	0
Sedation	121	87	34	242	1185
Seizures	196	34	12	242	1185
Constipation	139	53	13	205	1222
Bleeding	98	53	36	187	1240
Orthostatism	72	44	32	148	1279
Arrhythmia	85	40	11	136	1291
Anticholinergic	47	42	26	115	1312
Renal toxicity	114	0	0	114	1313
Serotonergic	31	18	18	67	1360

The risks for sedation and constipation were concluded to be both additive and dose-dependent. For anticholinergic or serotonergic side effects, orthostatism, and renal toxicity, the risks are additive and may occur even at ordinary doses. The bleeding risk has been shown to increase synergistically with the use of different types of anticoagulant drugs. As for QT prolongation and the risk of cardiac arrhythmia, it is still not known to what extent the risk adds up from different drugs, in the absence of a pharmacokinetic interaction that would markedly increase drug exposure. Several different mechanisms may be involved, as well as genetic variation in the susceptibility of individual patients. The cutoff level for a high risk from the summation of scores from two or more substances was set at 6 for sedation and serotonergic effects; 5 for orthostatism; 4 for constipation, bleeding risk, QT prolongation, and renal toxicity; and 3 for anticholinergic effects. For the risk of seizures, the highest individual score will determine the risk level, with no summation of scores.

### Pilot study registrations

During the study period, the PHARAO system was used 933 times in a total of 871 patients, 503 of which were treated in geriatric wards and 368 in primary care. Only the first PHARAO report for each patient was included for further analysis, with 55 out of 933 reports being excluded as “duplicates.” The mean number of medicines per patient analyzed by PHARAO was 14 in geriatric care and 8.6 in primary care. The mean ages of the patients were 84 years in geriatric care and 69 years in primary care, and 65 and 62%, respectively, were women.

**Fig. 2:**
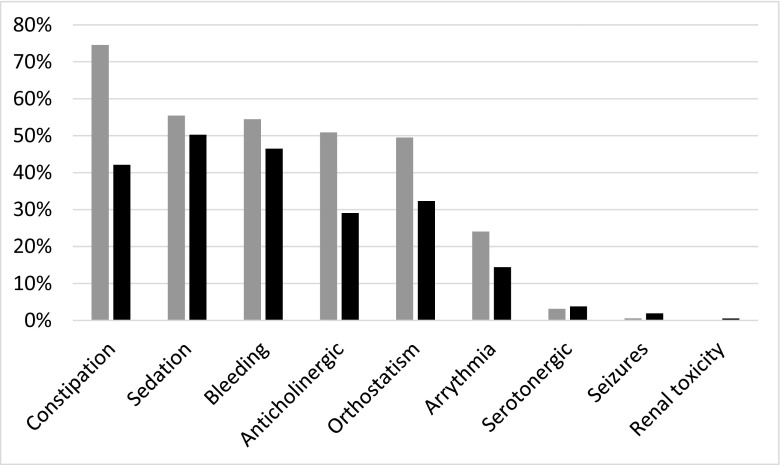
Frequency of the different PHARAO signals (medium- or high-risk) in relation to the medication list of geriatric patients (gray bars, *n* = 503) and primary care patients (black bars, *n* = 368)

**Fig. 3:**
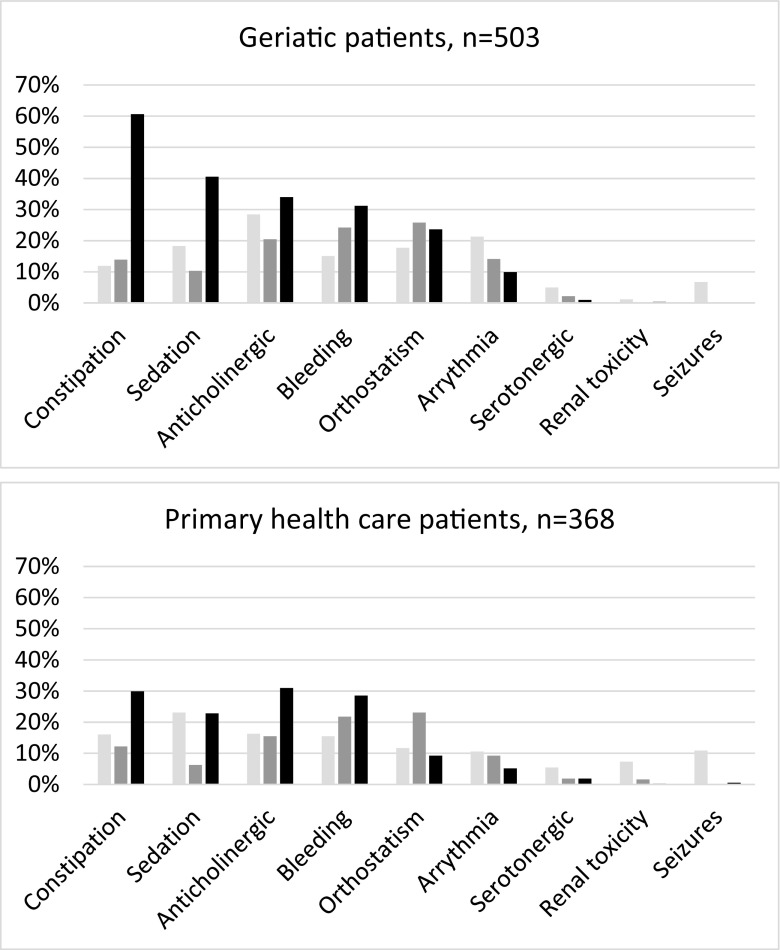
Frequency of PHARAO signals in relation to the medication list of geriatric and primary care patients. “Low-risk” signals in light gray bars, “medium-risk” signals in dark gray bars, and “high-risk” signals in black bars

The frequencies of the different risks, when adding medium- and high-risk signals in the two patient groups, are shown in Fig. 2. The most common signals were related to the risk of constipation, sedation, and bleeding, whereas only a few percent of patients had a risk signal related to serotonergic effects, seizures, or renal toxicity. The frequencies of all three risk levels in geriatric and primary care patients are shown in Fig. 3, with similar profiles in both groups, but somewhat lower risk frequencies in primary healthcare patients. The most frequent substances to cause a risk are listed in Table 2. Oxycodone, citalopram, and morphine were the three most commonly involved substances.

**Table 2:** The three most common substances leading to signals (score 1–3) for each of the risk properties in the pilot study

**Anticholinergic**	Oxycodone	Citalopram	Morphine
**Constipation**	Furosemide	Oxycodone	Bisoprolol
**Sedation**	Zopiclone	Oxycodone	Oxazepam
**Orthostatism**	Oxycodone	Glyceryl trinitrate	Morphine
**Bleeding**	Aspirin, low dose	Warfarin	Citalopram
**Serotonergic**	Citalopram	Metoclopramide	Mirtazapine
**Seizures**	Oxycodone	Citalopram	Morphine
**Arrhythmia**	Oxycodone	Citalopram	Salbutamol
**Renal toxicity**	Spironolactone	Naproxen	Diclofenac

### Pilot study questionnaires

Sixty-one physicians (32 from primary care and 29 from geriatric care) completed the pre-study questionnaire, corresponding to a 47% response rate. Two thirds of respondents were specialist (consultant) physicians. The group was equally distributed with respect to the number of years in practice, from less than 5 to more than 20 years. Primary care physicians answered that they would already often consider the risk of sedation, orthostatism, and renal toxicity in patients with several medicines. In addition, physicians in geriatrics often considered the risk of constipation. Primary care physicians stated that they would often meet patients suffering from constipation or bleedings as side effects, whereas geriatricians rated constipation, orthostatism, and renal toxicity as the most common side effects among their patients. Other important drug-related problems mentioned included compliance issues, incorrect medication lists, and involvement of many caregivers. When asked about their expectations on the PHARAO system, both groups expressed a hope that they would easily and quickly get an overview over important drug-related risks and recommendations on alternative treatment strategies. Geriatricians stressed their expectations to be able to perform medication reviews in a more structured way and of higher quality.

**Fig. 4:**
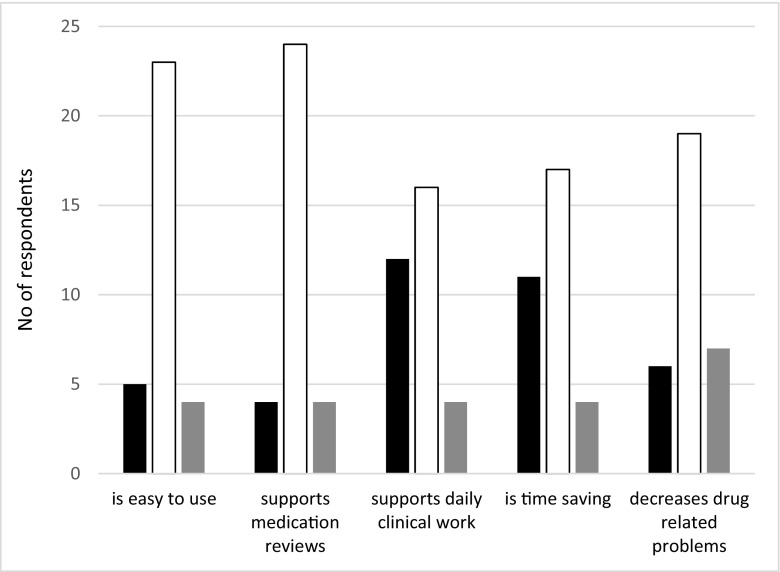
Rating of the usability of PHARAO by pilot test users (17 primary care physicians and 15 geriatricians) on a scale of 1–6. No. of responses with low grades (1–3) in black bars and high grades (4–6) in white bars. Gray bars represent “do not know/cannot answer”

The second questionnaire, after the study period, was answered by 40 out of 119 physicians, corresponding to a response rate of 33%. Eight of the respondents had not used PHARAO during the test period, three of which had not worked with patients, three had forgotten about it, and two stated no need for the tool. The following results are based on the answers from 32 respondents (17 primary care physicians and 15 geriatricians). In grading the general usefulness of PHARAO on a scale from 1 to 6, the mean grading was 3.7. A majority of physicians considered PHARAO to be easy to use and that it gives useful support in performing medication reviews. The scoring was somewhat lower for “support in daily work” and “time-saving” (Fig. 4). One responder commented on the time saving aspect, “The reason the system does *not* save time is that I received new information regarding my patients that I had to deal with.” Three respondents had learned a lot about side effect risks, 19 had learned a little, and 5 each had not learned anything new or could not answer that question. Twenty-one of the respondents would recommend PHARAO to a colleague, another five would recommend the system with modifications, and one would not recommend it.

## Discussion

The PHARAO system was designed to function as a complement to the Sfinx drug-drug interaction database, as a computerized clinical decision support system providing information on the risk of some common and/or serious adverse events, associated with a summation of pharmacodynamic effects from multiple medicines. We are not aware of any similar system, combining information on paired drug-drug interactions with summation effects from several medicines. There are several other types of computerized decision support systems to improve polypharmacy in the elderly. The Janus toolbar, produced by the Department of E-health and Strategic IT, Stockholm County Council, already includes functions signaling for drug-drug interactions (Sfinx database), dosing according to renal function, and warnings for inappropriate drugs for the elderly. Alagiakrishnan et al. describe the development and testing of the SMART system, based on Beer’s criteria for medication management in the elderly, combined with an estimation of renal function [20]. The Drug Burden Index Calculator was designed as a support tool for pharmacist-led medication reviews, to evaluate the total effect of anticholinergic and sedative properties from a medication list, including information on dosages [21, 22]. In a study of the views of general practitioners on the usefulness of the STOPP and START criteria [23] to improve pharmacological treatment of the elderly, it was concluded that the most important adaptation would be the development of a computerized version [24].

A decision support system is composed of several parts: a knowledge database, algorithms and integration rules (scripts) for combining database and patient information, and finally, an interface presenting the result to the end user. The integration and the presentation aspects of the decision support system are as crucial for the functioning of the system as is a reliable, validated and updated knowledge database [12, 25]. Pilot testing of a decision support system is an important part of the development process. One limit of this pilot study was the rather low response rate to the questionnaires, yet there were some important lessons to be learnt. Through the integration process, a medicine given in two tablets of different strength (i.e., morphine 10 mg plus morphine 5 mg) would count as two medicines, with added risks. Furthermore, all topical administration forms were included, irrespective of whether they have systemic effects or not. Beta-2 receptor agonists were classified as having a score of 1 in the risk of QT prolongation. This risk has since been re-evaluated as not clinically relevant in this context and removed. These features lead to a significant increase of “unnecessary” signals, and this was commented upon by the users. The PHARAO system does not include information about doses. Hence, the end user will not get a signal for an unnecessarily high dose of a single substance. This is a limitation, and an area for future development.

On the whole, the risks of constipation, sedation, and anticholinergic adverse effects were the most common adverse effects in both geriatric and primary healthcare patients in this study. These symptoms are important quality-of-life aspects in old age and may deserve more attention. A signal from the PHARAO system may possibly serve as a reminder to discuss these symptoms with the patient and their possible relation to drug treatment.

The increased risk of bleeding as a result of multiple drug use was also evident in both geriatric and primary care patients. Here, some physicians may be unaware of antithrombotic properties of drug groups, such as serotonin reuptake inhibitors or of the synergistic action of several antithrombotic drugs [26].

PHARAO may also be seen as a differential diagnostic tool to analyze whether the existing symptoms of the patient are medication- or disease-related, rather than a diagnosis-making tool, in which it could induce unnecessary clinical interventions.

Decision support systems need to be tested and evaluated like any other healthcare interventions. Preferably, there should also be studies on healthcare outcomes in relation to the use of such systems. For the PHARAO system, further studies on the relation between signals generated and actual symptoms in the patients are warranted.

## Conclusion

Clear descriptions of the content and functioning of computerized decision support systems, as well as pilot testing and continuous development, are important components to support the rational use and efficiency of the systems. The PHARAO decision support system, designed as a complement to the Sfinx database on drug-drug interactions, worked as intended and was appreciated by the users during a 4-month test period. Integration aspects need to be improved to minimize unnecessary signaling and thus increase the usefulness of the system.
